# Computational Study of Intramolecular Heterocyclic Ring Formation with Cyclic Phosphazenes

**Published:** 2014-09-06

**Authors:** Whelton A. Miller, Preston B. Moore

**Affiliations:** Department of Bioengineering, School of Engineering and Applied Science, University of Pennsylvania, Philadelphia, PA 19104 USA; Department of Chemistry and Biochemistry, University of the Sciences in Philadelphia, Philadelphia, PA 19104 USA

**Keywords:** Phosphazenes, Cyclic Phosphazenes, Ring Formation, Quantum Mechanics (QM), Density Functional Theory (DFT), Phosphorus Nuclear Magnetic Resonance (^31^P NMR), Becke Lee, Yang, Parr hybride Density Functional Theory (B3LYP)

## Abstract

Polyphosphazenes, because of their unique properties, have generated many opportunities to explore a variety of applications. These applications include areas such as biomedical research (e.g. drug delivery) and material science (e.g. fire-resistant polymers). Phosphazenes potentially have more variations then benzene analogues because of different substitution patterns. Here we present A computational study of the chemical modifications to a group of cyclic phosphazenes mainly hexachlorophosphazene (PNCl_2_)_3_. This study focuses on the relative energies of reactivity of hexachlorophosphazene to understand their geometry and the complexes they likely form. We compare diols, amino alcohols, and diamines with a carbon linker of 1-7 atoms. These heteroatom chains are attached to a single phosphorus atom or adjoining phosphorus atoms to form ring structures of geminal, vicinal (cis), and vicinal (trans) moieties. We find that the reactivities of “heteroatom caps” are predicted to be O,O (diol) > N,O (amino alcohol) > N,N (diamine). These results can be used to predict energetics and thus the stability of new compounds for biomedical and industrial applications.

## I. Introduction

**Fig 1:**
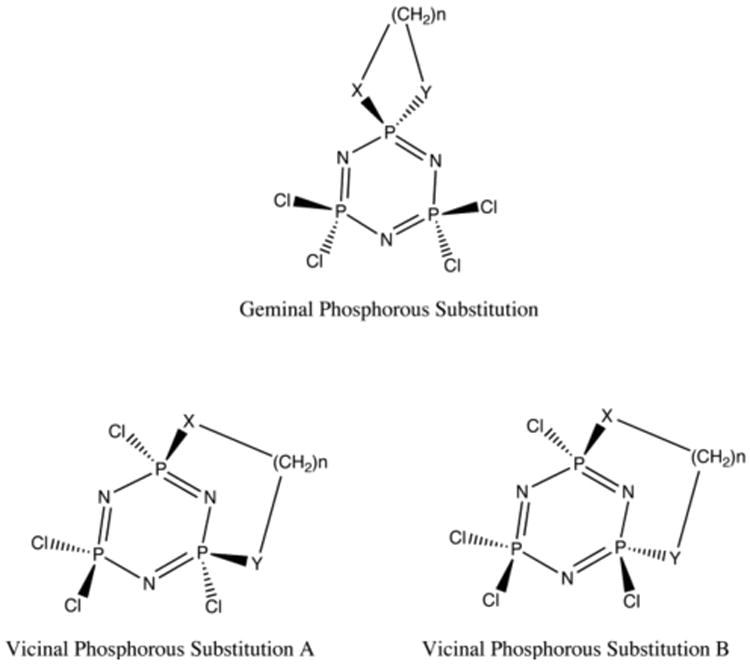
Substitution patterns for cyclized phosphazenes. X and Y are heteroatoms, i.e., N or O.

This Polyphosphazenes have been used in a variety of application, including several areas of biomedical research, such as drug delivery systems [1,2], and in material science, such as fire-resistant polymers [3]. Due to their substituition patterns, phosphazenes potenially have more variations than benzene analogs. Here we present a computational study of the chemical modifications to a group of cyclic phosphazenes (figure 1). We report the general conformation of the compounds, as well as relative energies, and potential applications.

In the past, phosphazenes have been studied for their properties both as linear polymers [4,5,6], and cyclic structures [7,8,9]. Their general electronic structure has also been studied [10,11,12], focusing on the interactions between the nitrogen and phosphorus atoms linear or cyclic geometries. Intramolecular reactions of difunctional nucleophiles, amino-alcohols, diols, and diamines with non-substituted cyclic phosphazenes such as hexachlorophosphazene (NPCl_2_)_3_, can yield three types of products [13-16]. First, the dinucleophile may replace two chlorine atoms at the same phosphorus atom to form geminal or spirocyclic structures [17-21]. Second, chlorine units on adjacent phosphorus atoms may be replaced to produce a vicinal substituted transannular-bridged *cis*-structure [20,21]. Third, substitution can produce a *trans*-vicinal bridge (figure 1) [13,20].

In this paper we focus on the relative energies of hexachlorophosphazenes with heteroatom chains, specifically the energy difference between geminal and vicinal structures. By using Quantum Mechanics (QM) to calculate the energy of formation of the resulting geminal and vicinal substituted structures, we strive to understand the relationship between heteroatom chain length, the resulting cyclized compound, and the likelihood of compound formation.

Phosphorus Nuclear Magnetic Resonance (^31^P NMR) spectroscopy is widely used to investigate the structure of phosphorus compounds. We use QM calculations to predict the ^31^P NMR shifts of our structures, and therefore correlate structural changes to changes in shielding. We analyzed our results, including the ^31^P NMR predictions, to further understand the relative energies of dinucleophiles with cyclic phosphazenes. Our analysis allows the prediction of reactivity and the rational design of future reactions and materials based on phosphazene chemistry.

## II. Methodology

**Scheme 1:**
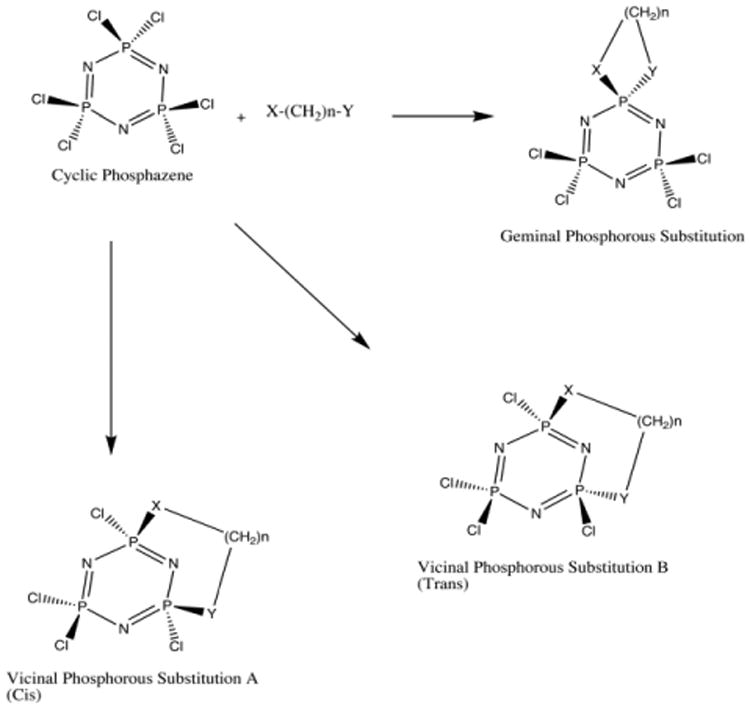
Reaction schemes for cyclic phosphazene with Heteroatom chains. X and Y are heteroatom “caps,” i.e., N or O for geminal substitution, vicinal (*cis*) and vicinal (*trans*) substitution.

We examined three distinct conformations, consisting of geminal (spirocyclic) and vicinal (transannular) bridged compounds (*cis* or *trans*) (figure 1). We attached heteroatom chains 1-9 atoms in length, including heteroatom caps, but excluding hydrogen (H_a_X-(CH_2_)_n_-YH_b_), where X and Y can consist of any combination of O or N. Conformational analysis on the expected products was performed with Molecular Operating Environment (MOE) [22]. The geometry was optimized using Hartree Fock (HF), and Becke Lee, Yang, and Parr hybrid Density Functional Theory (DFT) B3LYP with basis set 6-31g(d,p), and 6-31+g(d,p) [23-25]. Comparative calculations were done under tight optimization parameters, with diffusion functions added to the calculation to account for the possible interaction of the lone pair electrons on N, O, P, and Cl atoms. Calculations were carried out using Gaussian03 [26]. The lowest energy structures after optimization are reported and used for analysis. We then used equation 1 to determine the reaction energy (ΔE), allowing for discrimination between favorable and unfavorable reactions (scheme 1). ^31^P NMR calculations were performed with the GIAO [26,27] method from the Gaussian03 package, using DFT with basis set 6-31 g(d,p).

(1) $$\begin{array}{c} (A)\;E_{\left(\text{final}\right)}=E_{\left(\text{PhosphazeneFP}\right)}+E_{\left(2\text{HCl}\right)}\\ (B)\;E_{\left(\text{initial}\right)}=E_{\left(\text{Phosphazene ring}(P3N3\text{Cl}6)+\text{Amino Alcohol}\right)}+E_{\left(\text{HaX}-(\text{CH}2)n-\text{YHb}\right)}\\ (C)\;\Delta E_{\left(\text{reaction}\right)}=E_{\left(\text{final}\right)}-E_{\left(\text{initial}\right)} \end{array}$$

## III. Results and Discussion

When compared to the cyclic phosphazenes, carbon analogs such as benzene are limited because they permit only single substitution at a given position on the ring. Due to the substitution pattern of phosphazenes, branching can be significantly different than in carbon analogs. One such substitution pattern is geminal substitution. The geminal “spirocyclic” structures form a bicyclic ring at the phosphorous. Vicinal structures consist of attachments at two adjacent phosphorous atoms that can be either *cis* where the attachment is on the same side of the ring, or *trans* where the attachment is on different sides of the ring. In our investigations we will use a carbon chain capped with heteroatoms, where X and Y can be either N or O in any substitution pattern to react with the phosphazene (scheme 1). The result of a secondary intra-atomic reaction made after the initial heteroatom bond can give either the geminal or vicinal (*cis* or *trans*) substitution pattern.

### A. Geminal Cyclic Phosphazene Complexes

**Fig 2:**
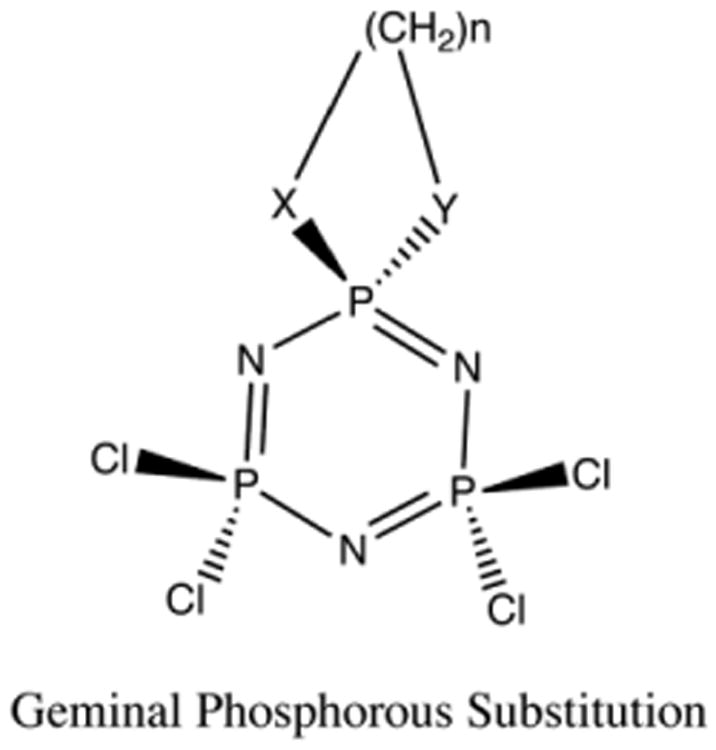
Geminal Substituted Cyclized Phosphazene. X and Y can equal N or O.

Geminal structures (figure 2) can be precursors to larger phosphazene macromolecules. These compounds in turn can be incorporated as dendrimers and linear polymers for numerous uses. Understanding the energetics of these systems, therefore, is crucial to understanding the structural properties, preferred products of reactions, and by extension, the properties and reactivity of larger phosphazene polymers.

**Fig 3:**
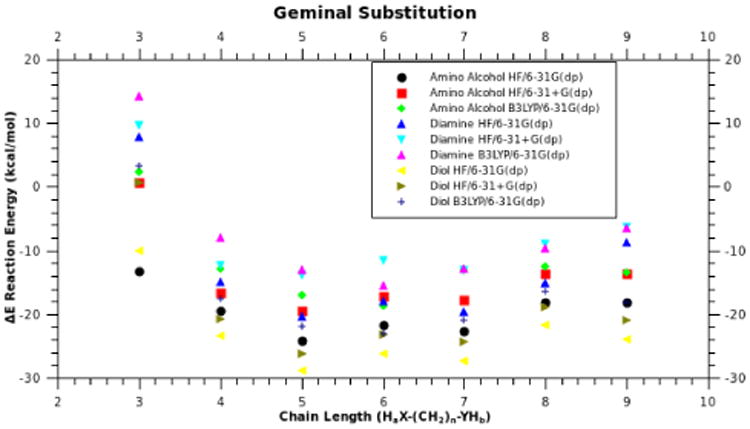
Plot of energy (ΔE) verses chain length for geminal cyclic phosphazenes. X-axis represents H_a_X-(CH_2_)_n_-YH_b_, where X and Y can be O or N. Each product has been calculated using three different levels of theory.

Figure 3 shows the energy of formation of geminal substituted phosphazenes. For each product we used three different levels of theory to assess basis set dependence, and observed that the trend is independent of the theory used. Specifically, the energy of formation is highly dependent on the ability to form cyclic rings and is lowest for the 5-atom (3-carbon chain capped with heteroatoms) chain that forms a six-membered ring, completed by the phosphorus atom. This preference parallels the well-known strain of ring formation in carbon analogs such as cyclohexane. The “dip” in energy at the 5-atom chain length is similar to the dip in energy seen in the formation of cyclohexane, when compared to cyclobutane, cyclopentane, cycloheptane, etc [27]. This can be seen in the energy difference between the 5-atom, 6-atom, and 7-atom chain lengths and the rest of the complexes. We find that for structures resulting in geminal substitution, the energy is almost solely dependent on the size and geometry of the resulting geminal ring with little influence from the phosphazene ring.

### B. Vicinal Bridged Cyclic Phosphazene Complexes

Reactions of difunctional nucleophiles with cyclophosphazenes to yield vicinal substitution patterns have been of considerable interest, even though they are found to be thermodynamically less stable compared to geminal or spiro-substituted cyclophosphazenes [29]. Vicinally substituted structures create strain in the phosphazene ring for example, as the ring tries to “bend” the P-N-P angle to accommodate a bond between the heteroatom chain and the adjacent phosphorus atom. The bend in the P-N-P angle is less pronounced in *cis*, which depends on the heteroatom chain's ability to reach across the “face” of the cyclophosphazene, than in *trans* where the heteroatom chain crosses the back face of the cyclophosphazene to reach the adjacent phosphorus.

**Fig 4:**
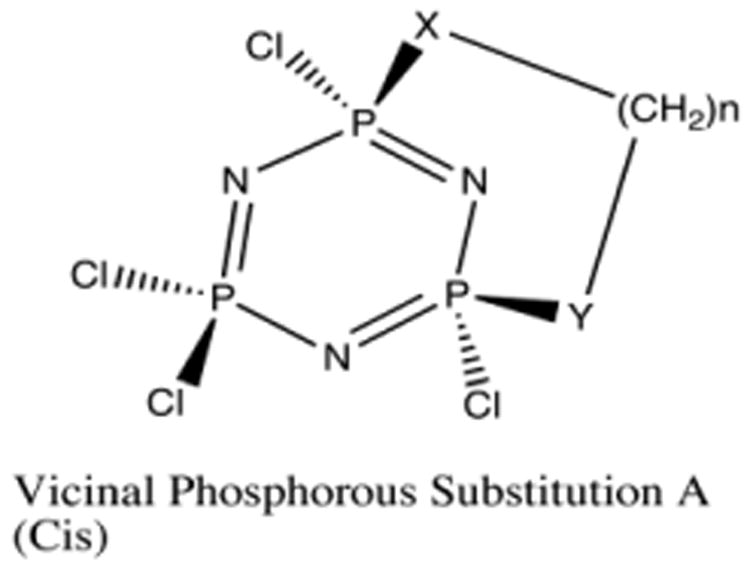
Vicinal Bridged (Cis) “Cis” Vicinal Substituted Cyclized Phosphazene, X and Y can equal N, or O. View of optimized geometries for “Vicinal (cis)” phosphazene complexes.

**Fig. 5:**
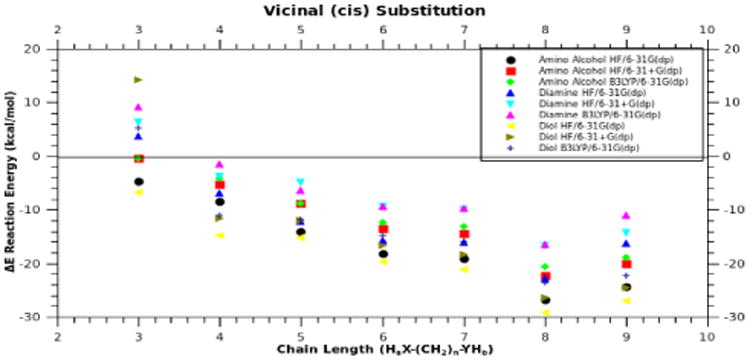
Plot of energy (ΔE) verses chain length for “*cis*” Vicinal substituted phosphazene. X-axis represents H_a_X-(CH_2_)_n_-YH_b_, where X and Y can be O or N. Each product was calculated using three different levels of theory.

As mentioned earlier, vicinal structures substituted in a *cis* (Figure 4) manner do not have a large additional ring strain arising from the carbon chain attempting to traverse cyclic phosphazenes. Without this “bending” at the P-N-P angle, the structures formed relate to chain length as expected. Favorable reactions begin at chain lengths of 4 atoms, depending on the heteroatom caps, with oxygen being more reactive (Figure 5). Again, we have used three different levels of theory to assess the basis set dependence on ΔE, and as before, the trends are independent of the basis set used. Chain lengths of 4 atoms or more are favored for any substitution pattern, diamines, diols, or amino-alcohols (i.e., ΔE)is < 0). There is about a ∼6-8 kcal/mol energy difference between the 7-atom chain length and the 8-atom chain length. As shown in Figure 5 the optimum chain length for vicinal (*cis*) substitution is 8 atoms long, which also happens to be the crossover point for vicinal (*cis*) and geminal substitution; both patterns have the same ΔE at this chain length (Figures 3 & 5). Although Figure 5 shows a consistent decrease in energy with an increase in chain length for 3-8 atoms, the 9-atom chain is slightly less favorable than the 8-atom chain. Initially, one might have expected the 9-atom chain to be more favorable because its greater number of degrees of freedom can accommodate ring constraints, but chain lengths larger than 8-atoms cause steric crowding at ring closure points, which accounts for the increase in energy for these lengths. We conclude that there are no geometries that will accommodate the constraints of the ring with an energy lower than that observed for 8-atoms chains.

**Fig. 6:**
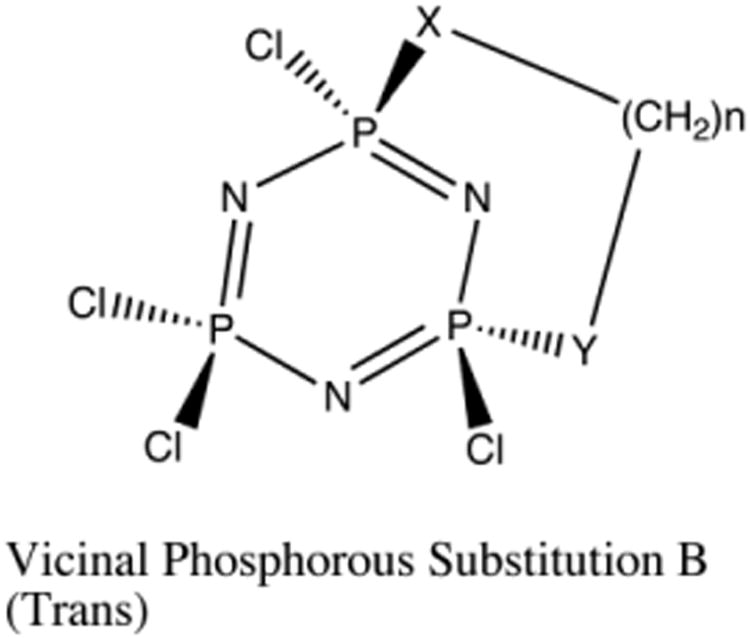
Vicinal Bridged (Trans) “Trans” Vicinal Substituted Cyclized Phosphazene. X and Y can equal N, or O.

**Fig. 7:**
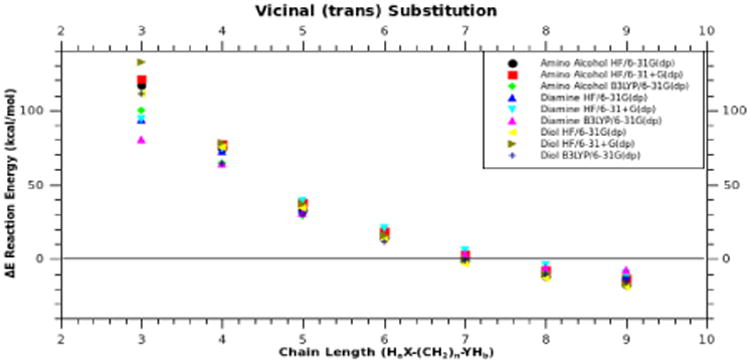
Plot of energy (ΔE) verses chain length for vicinal (*trans*) substituted phosphazene. X-axis represents H_a_X-(CH_2_)_n_-YH_b_, where X and Y can be O or N. Each product was calculated using three different levels of theory.

With respect to the geminal and vicinal (*cis*) compounds, vicinal (*trans*) substitution (Figure 6) is less dependent on the chain length or the ability to cyclize, but more dependent on the ring strain of the phosphazene (Figure 7). Ring strain breaks the planarity of the cyclic phosphazene, and decreases the “pseudo-resonance,” which is distinct from “full-resonance” in benzene [11]. The distortion of the phosphazene ring with respect to planarity adds a significant amount of energy to the formation energy of the resulting vicinal (*trans*) substituted phosphazene [30], which dominates the reaction energy of the heteroatom chain with the phosphazene. As expected, increased chain length reduces distortion, which favors reaction (Figure 7).

A comparison of Figure 5 to Figure 7 shows that *cis* substitution is favored over *trans* substitution in most cases. The energy difference between *trans* and *cis* vicinal substituted phosphazenes includes contributions from ring strain, and the ability of the chain to reach around the ring. We did find, however, that oxygen capped chains, i.e. diols and amino-alcohols, were more favored than diamines in vicinal (*cis*) and vicinal (*trans*) substitution.

### C. Comparison of Substitution Patterns

**Fig 8:**
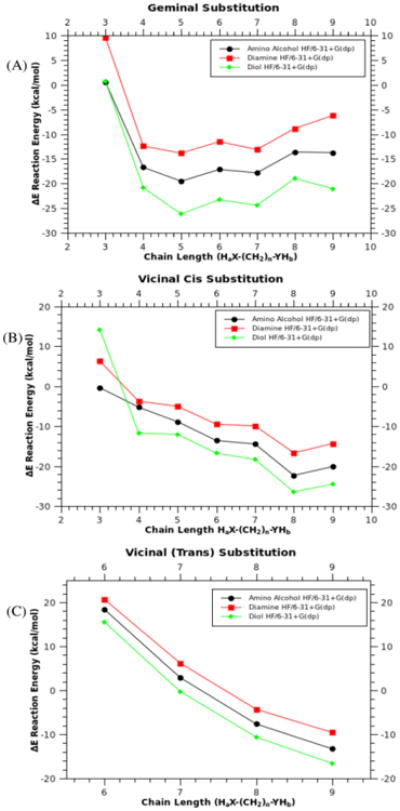
Plot of energy (ΔE) versus chain length for geminal (8a), vicinal (*cis*)(8b), vicinal (*trans*)(8c), and vicinal (*trans*, 6-9 atom chain length). X-axis represents H_a_X-(CH_2_)_n_-YH_b_, where X and Y can be O or N. Calculated using (HF/6-31+G(dp)).

As expected, in all cases oxygen serves as a stronger reactant than Nitrogen. Figure 8 shows that diols are more favorable, followed by the mixed heteroatom chains amino-alcohols, then diamines. Depending upon chain length, the second displacement will either add geminally, or *trans* or *cis* vicinally, with the geminal reactions being far more favorable.

**Fig. 9:**
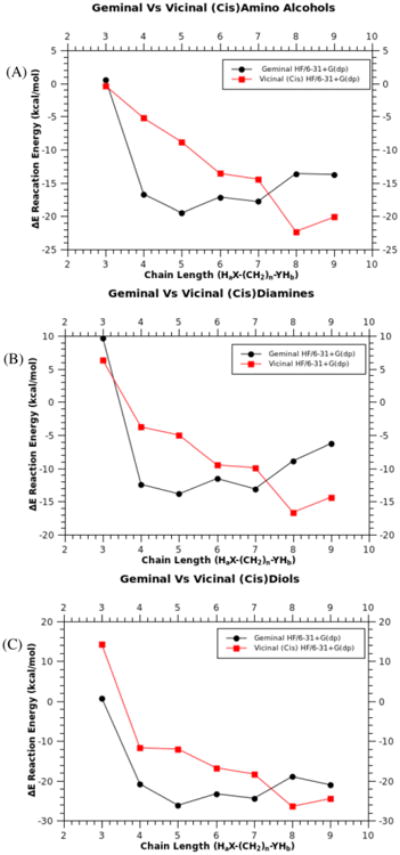
Plot of energy (ΔE) verses chain length for comparison of geminal versus vicinal (*cis*) substituted phosphazene, amino-alcohols (9a), diols (9b), and diamines (9c). X-axis represents H_a_X-(CH_2_)_n_-YH_b_, where X and Y can be O or N. Calculated using (HF/6-31+G(dp)).

**Fig 10:**
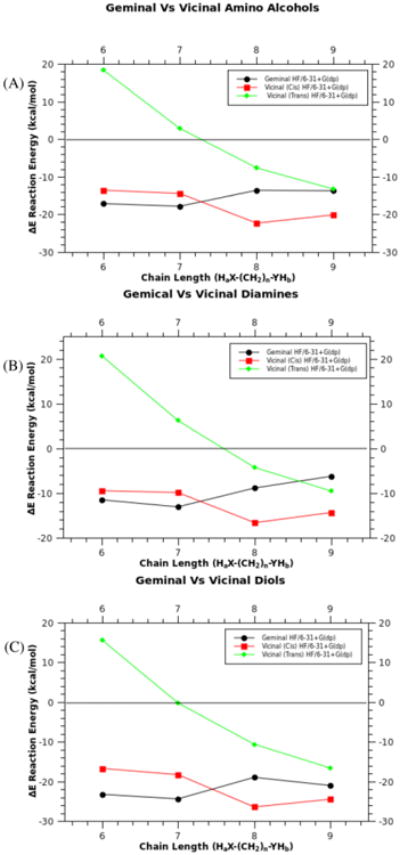
Plot of energy (ΔE) verses chain length and reaction energy for geminal versus vicinal (*cis* and *trans*) substituted phosphazenes, amino-alcohols (10a), diols (10b), and diamines (10c). X-axis represents H_a_X-(CH_2_)_n_-YH_b_, where X and Y can be O or N. Calculated using (HF/6-31+G(dp)).

Geminal addition is highly dependent upon the ability to form an intra-molecular ring, wth the stability of this ring being dependent upon its size. A conventional pattern of stability such as that seen in classic organic chemistry with respect to cycloalkane rings may be expected. For chain lengths ≤ 7 in all cases, diols, amino-alcohols, and diamines favor geminal substitution (Figure 9), with 5- and 6-membered rings being most stable. These rings are further stabilized by chair or boat conformation, with chair being prefered for six-membered geminal structures. Reactivity follows the previously observed trend (Figure 8) of diols first, followed by amino-alcohols and then diamines (Figures 9,10). Intra-atom reactions, which produce geminal substitution, are more favorable when compared to all vicinal substitutions at lower chain lengths.

Once the chain length increases beyond 5- and 6-membered rings, a “cross-over” of preference occurs due to steric interactions (Figure 9). These steric interactions are relieved by reacting with the adjacent (vicinal) phosphorous. Competition between *cis* and *trans* vicinal substitution is also dependent on steric interactions, although the distortion of the phosphazene ring plays a more important role (Figure 10). As the chain length increases and the ring strain decreases, the vicinal (*trans*) product becomes comparable in reactivity to the geminal and vicinal (*cis*) substituted products.

#### ^31^P NMR of cyclic Phosphazene structures

^31^P NMR has been widely used for the analysis of cyclophosphazenes and their analogs. Electronegative atoms can influence the phosphazene skeleton due to charge density at the neighboring phosphorus atoms [30-32]. Therefore, ^31^P NMR data can provide structural information while probing the nature of the electronic interaction within the phosphazene ring and any substituents. Electron withdrawal from the substituent-phosphorus bond can lead to either shielding or deshielding of the phosphorus atom. Substituent effects on phosphorus chemical shifts will depend on electronegativity and the bond angle of the substituent attached [31]. QM calculations determine the difference in shielding, which distinguishes between compounds substituted geminally or vicinally. Shielding can then be adjusted using further QM calculations with respect to a reference (330ppm, hexachlorophosphazene), or further adjusted from experimental data (21.28ppm, hexachlorophosphazene in CDCl_3_) for more accurate calculated spectra. Using this method we determined the degeneracy, relative chemical shifts, and neglect splitting patterns of these molecules. For geminal substitution, we expected that non-substituted phosphorus atoms (PCl_2_) would have similar chemical shifts, and this hypothesis was confirmed by the calculated ^31^P NMR. Because substitution only takes place on a single phosphorus atom, the chemical shift should only be reflected at that atom. Table 1 shows changes in shielding only in the PR_2_ phosphorus atom. We found similar chemical shifts in the two remaining PCl_2_ phosphorus atoms for geminally substituted diols, diamines, and amino-alcohols.

For vicinal (*cis*) molecules, the calculated ^31^P NMR shows similar chemical shifts for phosphorus atoms of like substitution (PClR). Becuase there is little or no disturbance to the planarity of the phosphazene ring system, distinction between phosphorus of like substitution is minimal. As expected, the two PClR phosphorus atoms showed approximately the same chemical shifts, while PCl phosphorus atoms had a significant difference in shielding.

For vicinal (*trans*) substitution, the expected result is similar chemical shifts at two of the three phosphorus atoms, similar to vicinal (*cis*). However, for compounds bridged with 3-5 atoms, the chemical shifts are different for all three phosphorus nuclei, which can be attributed to the ring distortion mentioned above. This distortion breaks the planarity of the cyclophosphorus ring and therefore disrupts the ring's “pseudo-resonance.” With this resonance disturbed, charge can become more localized, and therefore will affect the shielding of the local phosphorus atom. The vicinal (*trans*) phosphazene analogs affected by this distortion are in shown in italics (Table 3). This distortion effect occurs in products of short heteroatom chains; as chain length increases, distortion decreases similarly to the trend seen in Figure 7. Again, these observations indicate that ring distortion/loss of planarity of the phosphazene ring becomes less apparent as the chain becomes longer. Ring strain is no longer significant in chain lengths of 6 or more atoms.

## IV. Conclusion

The factors that influence the reactivity pattern of cyclic phosphazenes include the reactivity of a strong nucleophile, i.e. the basicity of the attaching reactant, and the steric influence of reactants and substituents already attached. The main influence highlighted by this work is steric hindrance by a bulky substituent. Based on the size and nature of the substituent, we can distinguish the different types of products expected.

(2) Reactivity=O−(CH2)n−O>N−(CH2)n−O>N−(CH2)n−N

(3) Geminial>Vicinal Cis>Vicinal Trans up ton=7.Atn=8vicinal cis>geminal

For diols, diamines, and amino alcohols, reactivity favors the oxygen-containing nucleophiles, diols, and amino alcohols over diamines (Equation 2, and Figure 8). This would be expected, because oxygen is a more electronegative nucleophile. Geminal and vicinal (*cis*) substitution is favored until chain length falls below 7 atoms. At chain lengths greater than 7, vicinal (*cis*) outcompetes geminal substitution (Equation 3).

Geminal substitution is dependent on its ability to form 5-or 6-membered rings, similar to carbon analogs such as cyclohexane. However, vicinal substitution is dependent on steric factors and the overall geometry of the ring. If the planarity of the phosphazene ring is distorted as seen in vicinal (*trans*) substitution, the ring distortion becomes the primary energy parameter. To avoid this ring distortion, the dinucleophillic chain must be able to reach the adjacent phosphorus atom. We found that chain lengths greater than 7 atoms have the best chance of avoiding this ring puckering phenomenon.

The reliance on longer chain lengths and the larger amount of steric interactions involved in vicinal substitution is why geminal substitution is favored for chain lengths greater than 7 atoms. *Cis* or *trans* substitution in vicinal compounds is, again, dictated by sterics. Steric hindrance from ring distortion versus the ability to reach across the “face” of the phosphazene distinguishes between vicinal (*cis*) and vicinal (*trans*) substitution. At chain lengths greater than 7, this hindrance becomes less of a factor, and trans substitution becomes competitive.

We have shown that ^31^P NMR can be used to track the substitution patterns and the differences between geminal, vicinal (*cis*), and vicinal (*trans*) substitution. Geminal substitution distinguishes a change only on a single phosphorus atom, which corresponds to substitution on a single phosphorus atom. For vicinal (*trans*), we found similar chemical shifts between the substituted phosphorus atoms at chain lengths n ≥ 4. At chain lengths n ≤ 3, we observed differences in shielding of all phosphorus nuclei due to a loss of resonance resulting from ring distortion. Vicinal (*cis*) shows similar chemical shifts for similarly substituted phosphorus atoms, as expected, and the ring distortion effect is negligible in these compounds. Changing the basis set does show a change in relative energy, however, the pattern of relative energy remains the same. In summary, we found that different properties can be created by varying heteroatom chain length. Using this information, we hope to contribute to future design of larger phosphazene polymers and other biomedical compounds. We believe that better understanding of the substitution patterns is critical to better design of these compounds.

## Figures and Tables

**Fig 11:**
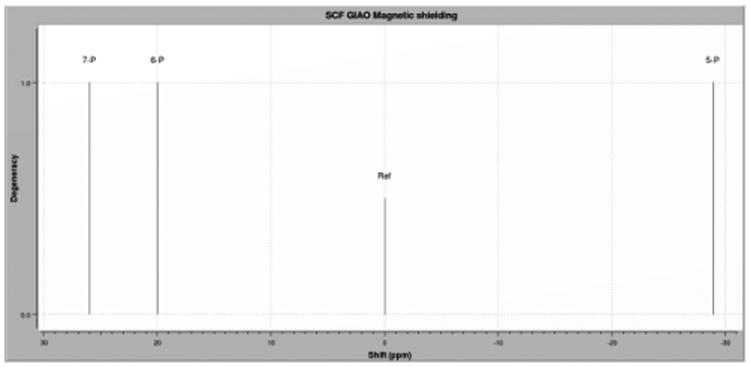
Geminal ^31^P data Calculated Isotropic ^31^P NMR spectra using hexachlorophosphazene as a reference (351.28ppm), for geminal substituted amino alcohol with 5-atom heteroatom chain. Calculated using DFT (B3LYP/6-31G(dp)).

**Fig. 12:**
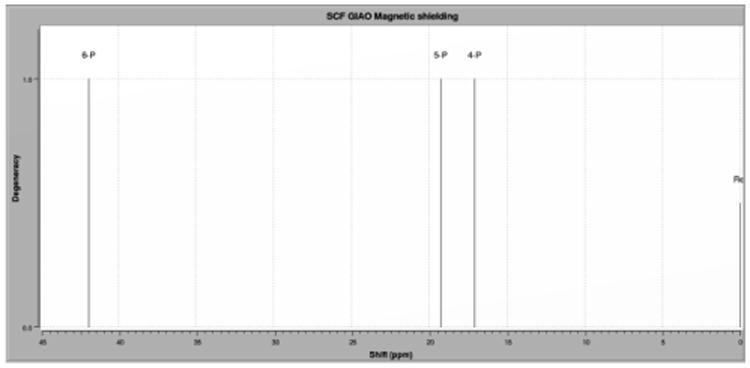
Vicinal (*cis*) ^31^P data Calculated Isotropic ^31^P NMR spectra using hexachlorophosphazene as a reference (351.28ppm) for vicinal (*cis*) substituted amino alcohol with 5-atom heteroatom chain. Calculated using DFT (B3LYP/6-31G(dp)).

**Fig. 13:**
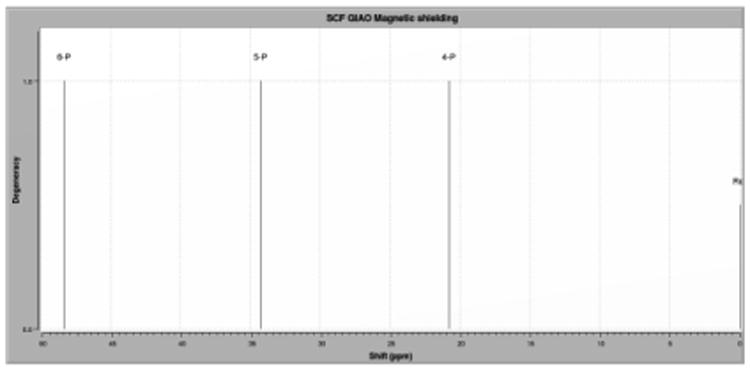
Vicinal (*trans*) ^31^P data Calculated Isotropic ^31^P NMR spectra using hexachlorophosphazene as a reference (351.28ppm), for vicinal (*trans*) substituted amino alcohol with 5-atom heteroatom chain. Calculated using DFT (B3LYP/6-31G(dp)).

**Tab. 1:** Calculated isotropic ^31^P NMR data using hexachlorophosphazene as a reference (351.28ppm), for geminal substituted amino alcohols, diamines, and diols by chain length. Calculated using DFT (B3LYP/6-31G(dp)).

X-(CH2)_n_-Y	O-(CH2)_n_-N			N-(CH2)_n_-N			O-(CH2)_n_-O		
Number of Atoms	PR_2_	PCl_2_	PCl_2_	PR_2_	PCl_2_	PCl_2_	PR_2_	PCl_2_	PCl_2_
3	-7.88	40.63	39.60	-11.20	37.36	38.55	-10.09	41.21	41.21
4	-6.07	39.64	40.19	-2.71	38.71	33.71	-3.34	40.15	40.15
5	-28.87	20.00	26.00	-18.35	35.71	39.84	-24.70	34.55	39.23
6	-25.23	22.16	25.88	-21.34	36.38	36.36	-16.63	37.28	37.30
7	6.24	35.21	35.62	-23.30	36.43	33.77	-21.09	37.23	35.44
8	-27.88	24.63	18.79	-22.41	35.29	35.85	-21.30	35.78	37.02
9	-29.37	20.67	26.19	-19.22	35.15	33.39	-21.32	36.77	33.62

**Tab. 2:** Calculated isotropic ^31^P NMR data using hexachlorophosphazene in CDCl3 as a reference (351.28ppm), for vicinal (*cis*) Substituted amino alcohols, diamines, and diols by chain length. Calculated using DFT (B3LYP/6-31G(dp)).

X-(CH2)_n_-Y	O-(CH2)_n_-N			N-(CH2)_n_-N			O-(CH2)_n_-O		
Number of Atoms	PClR (ppm)	PClR (ppm)	PCl_2_	PClR	PClR	PCl_2_	PClR	PClR	PCl_2_
3	29.53	24.16	38.48	28.00	28.00	34.98	27.16	27.16	42.41
4	32.32	21.87	45.50	28.60	22.20	43.27	33.93	23.37	45.21
5	17.09	19.25	41.89	17.45	17.45	39.86	15.12	21.26	41.17
6	14.24	15.11	39.62	15.85	15.43	38.52	14.11	11.06	40.51
7	10.55	12.18	38.11	11.59	13.20	36.61	12.39	8.76	39.01
8	12.84	12.87	39.59	14.16	4.70	41.91	11.45	13.57	41.50
9	11.88	16.13	41.65	4.88	18.13	42.26	14.07	8.40	42.44

**Tab. 3:** Calculated isotropic ^31^P NMR data using hexachlorophosphazene in CDCl3 as a reference (351.28ppm), for vicinal (*trans*) substituted amino alcohols, diamines, and diols by chain length. Calculated using DFT (B3LYP/6-31G(dp)). Those phosphorus atoms affected by ring puckering are shown in italics and bold type.

X-(CH2)_n_-Y	O-(CH2)_n_-N			N-(CH2)_n_-N			O-(CH2)_n_-O		
Number of Atoms	PClR (ppm)	PClR (ppm)	PCl_2_ (ppm)	PClR (ppm)	PClR (ppm)	PCl_2_ (ppm)	PClR (ppm)	PClR (ppm)	PCl_2_ (ppm)
3	41.23	***140.12***	87.96	40.05	***107.37***	48.22	42.62	***140.12***	90.59
4	22.80	***55.57***	51.81	29.03	***72.68***	64.84	35.26	***71.80***	70.02
5	20.81	***34.27***	48.31	26.09	***49.09***	60.13	33.79	***49.27***	64.40
6	15.53	10.10	38.62	24.50	23.80	50.35	28.38	21.37	53.89
7	4.94	4.77	34.11	17.83	17.03	45.12	17.69	17.26	49.53
8	7.23	4.02	29.81	14.34	14.34	41.33	18.44	17.69	44.88
9	-8.25	-0.29	34.57	10.78	18.03	39.83	14.04	14.96	43.68

## Synopsis

Phosphazenes have potentially more variations than benzene analogs because of their unique substitution patterns. This study focuses on the properties of phosphazenes, in particular hexachlorophosphazene, to understand their geometry and the relationship of preferred structure to reactivity. To compare the interactions, we tethered diols, amino alcohols, and diamines with a carbon linker of 1-7 atoms. These were attached to form ring structures of geminal, vicinal-*cis*, and vicinal-*trans* moities.

## References

[1] Ibim SA, Ambrosio AA, Larrier D, Allcock HR, Laurencin CT (1996). Controlled macromolecule release from poly(phosphazene)matrices. J Controlled Release.

[2] Cho SY, Allcock HR (2007). Dendrimers Derived from Polyphosphazene–Poly(propyleneimine) Systems: Encapsulation and Triggered Release of Hydrophobic Guest Molecules. Macromolecules.

[3] Allen JY, Allcock HR (1993). The Use of Phosphazenes as Fire Resistant Materials. J Fire Sciences.

[4] Allcock HR, Kwon S (1989). An ionically crosslinkable polyphosphazene: poly[bis(carboxylatophenoxy)phosphazene] and its hydrogels and membranes. Macromolecules.

[5] Allcock HR, Turner ML (1993). Ring expansion and polymerization of transannular bridged cyclotriphosphazenes and their spirocyclic analogs. Macromolecules.

[6] Allcock HR, Dudley GK (1996). Lower Critical Solubility Temperature Study of Alkyl Ether Based Polyphosphazenes. Macromolecules.

[7] Davies DB, Clayton TA, Eaton RE, Shaw RA, Egan A, Hursthouse MB, Sykara GD, Porwolik-Czomperlik I, Siwy M, Brandt K (2000). Chiral Configurations of Cyclophosphazenes. J Am Chem Soc.

[8] Richards PI, Steiner A (2004). Cyclophosphazenes as Nodal Ligands in Coordination Polymers. Inorg Chem.

[9] Allcock HR, Kellam EC (2002). Incorporation of Cyclic Phosphazene Trimers into Saturated and Unsaturated Ethylene-like Polymer Backbones. Macromolecules.

[10] Breza M (2000). The electronic structure of planar phosphazene rings. Polyhedron.

[11] Chaplin AB, Harrison JA, Dyson PJ (2005). Revisiting the Electronic Structure of Phosphazenes. Inorg Chem.

[12] Haddon RC (1985). Chem Phys Letters.

[13] Allcock HR, Turner ML, Visscher KB (1992). Synthesis of transannular- and spiro-substituted cyclotriphosphazenes: x-ray crystal structures of 1,1-[N3P3(OCH2CF3)4{O2C12H8}],1,3-[N3P3(OCH2CF3)4{O2C12H8}], 1,1-[N3P3(OCH2CF3)4{O2C10H6}], and 1,3-[N3P3(OCH2CF3)4}O2C10H6}]. Inorg Chem.

[14] Allcock HR (1972). Recent advances in phosphazene (phosphonitrilic) chemistry. Chem Rev.

[15] Allcock HR, Rutt JS, Parvez M (1991). Synthesis of cyclic phosphazenes with isothiocyanato, thiourethane, and thiourea side groups: x-ray crystal structure of N3P3(NMe2)3(NCS)3. Inorg Chem.

[16] Allen CW (1991). Regio- and stereochemical control in substitution reactions of cyclophosphazenes. Chem Rev.

[17] Iter (2007). Phosphorus–Nitrogen Compounds. 14. Synthesis, Stereogenism, and Structural Investigations of Novel N/O Spirocyclic Phosphazene Derivatives. Inorg Chem.

[18] Muralidharan K, Venugopalan P, Elias AJ (2003). Ansa versus Spiro Substitution of Cyclophosphazenes: Is Fluorination Essential for Ansa to Spiro Transformation of Cyclophosphazenes?. Inorg Chem.

[19] Gabino GA (1998). Synthesis of New Phosphazene High Molecular Weight Polymers Containing Functionalized and Optically Active Spirocyclic Groups. Macromolecules.

[20] Elias AJ, Twamley B, Shreeve JM (2001). Syntheses and Experimental Studies on the Relative Stabilities of Spiro, Ansa, and Bridged Derivatives of Cyclic Tetrameric Fluorophosphazene. Inorg Chem.

[21] (2011). Molecular Operating Environment (MOE), 2011.10.

[22] Becke AD (1993). A new mixing of Hartree–Fock and local density-functional theories. J Chem Phys.

[23] Lee CT, Yang WT, Parr RG (1988). Development of the Colle-Salvetti conelation energy formula into a functional of the electron density. Physical Review B.

[24] Dunning TH, Huzinaga PJ (1976). Modern Theoretical Chemistry.

[25] Frisch (2004). Gaussian03.

[26] Jemmis ED, Kiran B (1998). Aromaticity in X^3^ Y^3^ H^6^ (X = B, Al, Ga; Y = N, P, As), X^3^ Z^3^ H^3^ (Z = O, S, Se), and Phosphazenes. Theoretical Study of the Structures, Energetics, and Magnetic Properties. Inorg Chem.

[27] McMurray J (2000). Organic Chemsitry.

[28] Muralidharan K, Elias A (2003). Preparation of the First Examples of Ansa–Spiro Substituted Fluorophosphazenes and Their Structural Studies: Analysis of C–H…F–P Weak Interactions in Substituted Fluorophosphazenes. J Inorg Chem.

[29] Krishnamurthy SS, Woods M (1987). Nuclear Magnetic Resonance of Cyclophosphazenes.

[30] Allcock HR, Harris PJ, Nissan RA (1981). Organometallic phosphazenes: synthesis and rearrangement of propynyl- and propadienylcyclotriphosphazenes. J Am Chem Soc.

[31] Benson MA, Zacchini S, Boomishanker R, Steiner A (2007). Alkylation and acylation of cyclotriphosphazenes. Inorg Chem.

